# Accidental Rivaroxaban Intoxication in a 25-month-old Child Monitored by Thromboelastography: A Case Report

**DOI:** 10.24546/0100499694

**Published:** 2026-01-29

**Authors:** KEITA NAKANISHI, SHOTA YAMASAKI, YUNA WATANABE, KAORU NAKASHIMA, TOMOKO NISHIHARA, JUNKO FUJII, SHOHEI OHYAMA, HIROKI YOKOYAMA, NAOKI YOKOYAMA

**Affiliations:** 1 Department of Pediatrics, Akashi Medical Hospital, Hyogo, Japan; 2 Department of Anesthesiology, Akashi Medical Hospital, Hyogo, Japan

**Keywords:** Factor Xa inhibitors, Novel oral anticoagulants, Overdose, TEG® 6s, Home accidents

## Abstract

Rivaroxaban is an oral, selective, direct-acting factor Xa inhibitor with high affinity for the active site of factor Xa. Thromboelastography (TEG® 6s) has reportedly been used to evaluate coagulation function under rivaroxaban administration, especially in the perioperative period. To date, there are no reports in the literature about thromboelastography in children that have ingested a large amount of rivaroxaban. We describe the case of a 25-month-old girl who accidentally ingested a large dose of rivaroxaban and we followed up on the coagulation performance with TEG® 6s. When she came to our emergency room, there was no apparent bleeding. The patient was hospitalized for observation while undergoing monitoring using TEG® 6s. Approximately 20 h after taking the medication, all values of her blood tests including TEG® 6s returned to the normal range and the patient was discharged, without evidence of bleeding. This case highlights the potential usefulness of TEG® 6s for evaluating the effects of rivaroxaban in pediatric accidental ingestion.

## INTRODUCTION

Rivaroxaban is an oral, selective, direct-acting factor Xa inhibitor with high affinity for the active site of factor Xa (1). Non-vitamin K antagonist oral anticoagulants, including rivaroxaban, are widely used to prevent thromboembolic diseases, especially in patients with atrial fibrillation (1).

Prothrombin time (PT) and activated partial thromboplastin time (aPTT) are commonly used as coagulation monitoring tests. However, point-of-care hemostatic coagulant monitoring using blood elasticity viscosity tests such as thromboelastgraphy (TEG® 6s) is reportedly useful, especially in the perioperative period, when more accurate information in a shorter time is needed (2). To date, there are no reports in the literature about TEG® 6s in children that have ingested a large amount of rivaroxaban.

We report the case of a child who accidentally ingested a large dose of rivaroxaban. We followed up on the coagulation performance with TEG® 6s.

## CLINICAL CASE

A 25-month-old girl (bodyweight: 13.3 kg) with atopic dermatitis was suspected to have ingested six 15 mg rivaroxaban tablets (total: 90 mg). She had no notable medical history or family history of congenital coagulation disorders such as hemophilia. On presentation, her general condition was good, and there were no signs of bleeding. Her appearance was good, work of breathing was normal, and circulation to the skin was good and without cyanosis. Vital signs were: temperature 37.6μ, heart rate 123 bpm and SpO_2_ 97%. She was alert, and Glasgow coma scale score was E4V5M6. There were no signs of unusual injuries throughout her body.

Considering the risk of massive bleeding, intravenous infusion of extracellular fluid was immediately started, and blood tests were performed at approximately 2 h after the suspected ingestion. Blood test results were: Hb 12.0 g/dL, platelets 36,8000/μL, PT 31.6 sec PT-INR 2.74 aPTT 67.9 sec (normal 24–37 sec). Considering the time to peak effect, the PT PT-INR aPTT was retested at 3 h post-dose, and a TEG® 6s test was performed. The results were: PT 30.4 sec, PT-INR 2.64, aPTT 64.3 sec (normal 24–37 sec), CK-R 29.0 min (normal 4.6–9.1 min), CFF-MA 19.5 mm (normal 15–32 mm), CRT-MA 67.0 mm (normal 52–70 mm).

Blood test results at approximately 2 and 3 h after ingestion indicated that the anticoagulant effect had peaked out. The patient was hospitalized for observation without specific treatment.

Approximately 20 h after taking the medication, the patient accidentally bumped her face, but there was no obvious bleeding. Blood tests, including TEG® 6s, were performed around the same time, and all values were within normal limits (Table I, Figures 1A and 1B) (Hb 12.6 g/dL, PT 12.5 sec PT-INR 1.10, aPTT 30.2 sec, CK-R 9.4 min, CFF-MA 19.3 mm, CRT-MA 63.1 mm). The patient was discharged, without evidence of bleeding.

**Figure 2:**
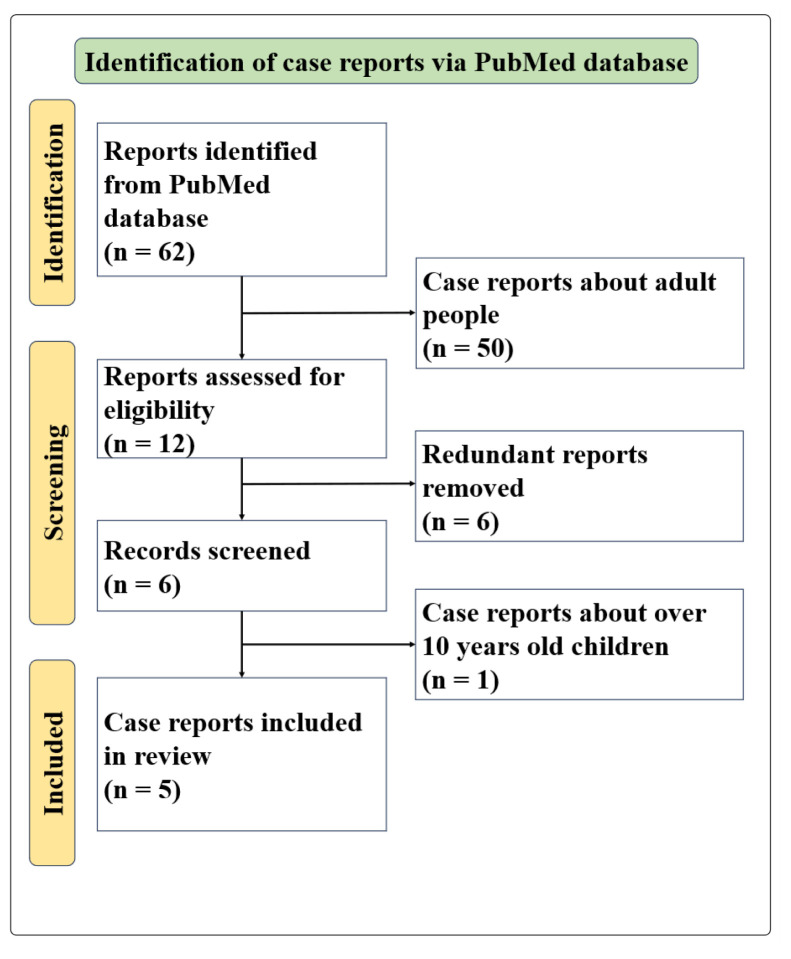
Flow diagram of the literature review

We performed a literature search to identify case reports of children who ingested a large amount of anti-factor Xa drugs, including rivaroxaban. Searches were performed using PubMed published up to October 2025. The search terms included combinations of; “direct acting oral anticoagulant,” “rivaroxaban,” “apixaban,” “dabigatran,” “edoxaban,” “overdose,” and “intoxication.” Case reports about children under 10 years old were included (Figure 2). From the literature reviews of anti Xa inhibitor ingestion in children (Table II) (3–7), five case reports indicate that although all pediatric patients exhibited transient prolongation of PT and aPTT in laboratory tests, none experienced significant bleeding symptoms. Regarding treatment, two patients were managed with observation without specific treatment, while some patients received administration of activated charcoal or Fresh Frozen Plasma.

## DISCUSSION

This patient accidentally ingested a large amount of rivaroxaban, but showed no signs of bleeding. We therefore managed her with careful in-hospital observation without specific therapy. Although ingestion was not directly witnessed, abnormal coagulation results (PT, aPTT, TEG® 6s) strongly supported rivaroxaban intake. According to reports on rivaroxaban, the peak time is 2–4 h and the half-life is approximately 8 h (8), which is generally consistent with our patient’s blood test results.

For patients at high risk of bleeding under rivaroxaban therapy, guidelines recommend reversal agents (such as andexanet alfa) or nonspecific hemostatic agents (such as prothrombin complex concentrate) (9).

However, clinical evidence remains limited, and pediatric cases in particular are rarely reported, so there is little guidance for appropriate management (9). In this case, no signs of active bleeding were observed after the expected peak effect, so we adopted a conservative policy of observation, and coagulation parameters normalized without complication. If bleeding had occurred, our plan was to administer andexanet alfa, which was available in our hospital.

TEG® 6s has been reported as a useful adjunct for evaluating coagulation under administration of rivaroxaban (2). Accordingly, we performed TEG testing in parallel with PT and aPTT, and both approaches were informative for assessing anticoagulant effect in this case. Although not applicable in this case because no severe bleeding occurred, in the event of severe bleeding with thromboelastography results strongly suggesting anticoagulant poisoning in addition to PT and aPTT findings, TEG testing could be crucial when deciding to administer specific antidotes such as andexanet alfa.

This case showed the potential usefulness of TEG® 6s for evaluating the anticoagulant coagulation effects from rivaroxaban in pediatric accidental ingestion. This report has several limitations. First, ingestion was not witnessed, so the exact timing and amount of rivaroxaban taken cannot be definitively confirmed. However, under the supervision of the patient’s grandparents, the number of empty medication packages could be accurately counted, allowing a reliable estimate. Second, plasma rivaroxaban concentration was not measured. Such data would have confirmed the ingestion and dose, and allowed direct comparison with cases in previous reports (6).

In conclusion, we described the case of a 25-month-old girl who ingested rivaroxaban but recovered uneventfully without specific treatment. Evaluation of coagulation function by TEG® 6s was thought to be helpful.

## Figures and Tables

**Figure 1A:**
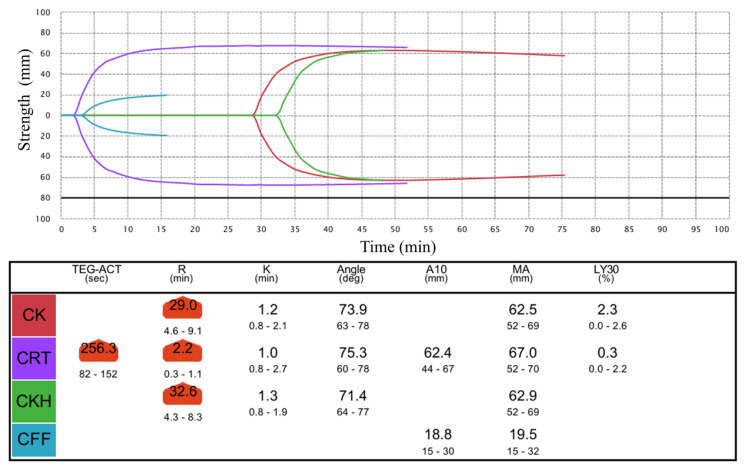
TEG® 6s curves at 3 hours after accidentally ingesting 90 mg of rivaroxaban CK, Citrated kaolin; CRT, Citrated rapid TEG®; CKH, Citrated kaolin heparinase; CFF, Citrated functional fibrinogen; ACT, activated clotting time; R, reaction time; K, coagulation time; A10, amplitude at 10 minutes; MA; maximum amplitude; LY30, lysis 30 minutes after maximum amplitude.

**Figure 1B:**
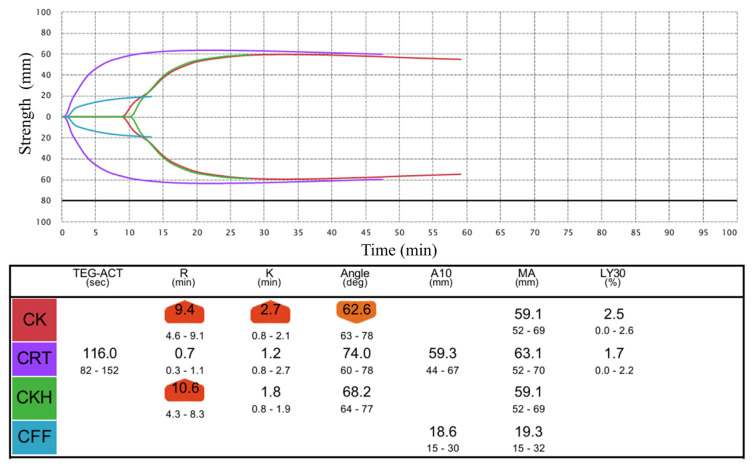
TEG® 6s curves at 20 hours after accidentally ingesting 90 mg of rivaroxaban CK, Citrated kaolin; CRT, Citrated rapid TEG®; CKH, Citrated kaolin heparinase; CFF, Citrated functional fibrinogen; ACT, activated clotting time; R, reaction time; K, coagulation time; A10, amplitude at 10 minutes; MA; maximum amplitude; LY30, lysis 30 minutes after maximum amplitude.

**Table I:** Variation of PT (%), aPTT (sec), TEG® 6s of a 25-month-old child at different times after accidentally ingesting 90 mg of rivaroxaban

	Time after ingesting (hours)		

	2	3	20
PT (%)	20	21	84
aPTT (sec)	67.9	64.3	30.2
CK-R (min)		29.0	9.4
CFF-MA (mm)		19.5	19.3
CRT-MA (mm)		67.0	63.1

CK-R, citrated kaolin-reaction time; CFF-MA, citrated functional fibrinogen maximum amplitude; CRT-MA, citrated RapidTEG maximum amplitude.

**Table II:** Published case reports of overdoses of Factor Xa inhibitors rivaroxaban and apixaban

Age (months old)	Body weight (kg)	Time to presentation	Amount of dosage	Co-ingestion	Tests performed	Treatment	Bleeding	Reference
35	12.5	15 minutes	Rivaroxaban 200 mg	None	Anti-FXa >4.00 IU/mL	AC	None	Carr et al. (2018)
18	Unknown	2 hours	Rivaroxaban Unknown (5 or 6 tablets)	None	PT 75.3 sec; INR 2.74; PTT 75.8 sec	FFP	None	Weirthein et al. (2019)
23	12.9	2 hours	Apixaban 40 mg	Digoxin 0.75 mg	Apixaban 1712 μg/L	None	None	Launey et al. (2020)
8	11	2 hours	Rivaroxaban 15 mg	None	Anti-Xa activity 4.72 IU/mL; PT INR 2.5; PTT 102 sec	AC	None	Ha et al. (2022)
18	13.1	2 hours	Apixaban 40 mg	Candesartan 24 mg	PT 38.7 sec; INR 3.3; aPTT 53 sec	None	None	Wiggins et al. (2024)

Anti-FXa, anti-factor Xa; IU, international units; AC, activated charcoal; PT, prothrombin time; INR, international normalized ratio; PTT, partial thromboplastin time; sec, seconds; FFP, fresh frozen plasma; aPTT, activated partial thromboplastin time.
